# Some aspects of entomological determinants of *Phlebotomus orientalis* in highland and lowland visceral leishmaniasis foci in northwestern Ethiopia

**DOI:** 10.1371/journal.pone.0192844

**Published:** 2018-02-13

**Authors:** Esayas Aklilu, Ibrahim Abbasi, Araya Gebresilassie, Solomon Yared, Mizan Kindu, Oscar David Kirstein, Aviad Moncaz, Habte Tekie, Meshesha Balkew, Alon Warburg, Asrat Hailu, Teshome Gebre-Michael

**Affiliations:** 1 Department of Biology, Mada Walabu University, Bale-Robe, Ethiopia; 2 Department of Microbiology and Molecular Genetics, The Institute of Medical Research Israel-Canada The Kuvin Center for the Study of Infectious and Tropical Diseases, Faculty of Medicine, The Hebrew University, Hadassah Medical School, Jerusalem, Israel; 3 Department of Biology, Jigjiga University, Jigjiga, Ethiopia; 4 School of Medicine, Parasitology, Microbiology and Immunology, Mada Walabu University, Bale-Robe, Ethiopia; 5 Department of Zoological Sciences, Addis Ababa University, Addis Ababa, Ethiopia; 6 Aklilu Lemma Institute of Pathobiology, Addis Ababa University, Addis Ababa, Ethiopia; 7 Department of Microbiology, Immunology and Parasitology, College of Health Sciences, Addis Ababa University, Addis Ababa, Ethiopia; Institut Pasteur, FRANCE

## Abstract

Visceral leishmaniasis (VL) is one of the major public health problems in northwest Ethiopia, mainly in Libo-Kemkem and Metema districts, where *Phlebotomus orientalis* is the most probable vector of the disease. The aim of this study was to determine the physiological age, host preference and vectorial potential of *P*. *orientalis* in the highland and lowland foci of the region. Sand flies were collected using CDC light traps between May 2011 and April 2012 in Libo-Kemkem and October 2012 and September 2013 in Metema from household compounds, farm field and mixed forest. Females belonging to *Phlebotomus* were dissected for physiological age determination and *Leishmania* detection and isolation. *Leishmania* infections in sand flies were investigated using molecular methods. Freshly fed *Phlebotomus* females were tested to identify blood meal sources using PCR-RLB and ELISA. A total of 1149 (936 from Libo-Kemkem and 213 from Metema) blood unfed female *P*. *orientalis* were dissected for age determination. The parity rate was 45.6% and 66.2% in Libo-Kemkem and Metema, respectively. None of 798 female *P*. *orientalis* dissected (578 from Libo-Kemkem and 220 from Metema) was infected with *Leishmania* parasites. A total of 347 *P*. *orientalis* specimens collected from Libo-Kemkem were processed using PCR, of which 10 (2.8%) specimens were found with DNA of *Leishmania* spp. Of a total 491 freshly fed female *P*. *orientalis* analyzed for blood meal origins by RLB-PCR and ELISA, 57.6% (67.8% from Libo-Kemkem and 49.8% from Metema) were found to contain bovine blood while 4.9% (3.7% from Libo-Kemkem and 5.7% from Metema) were of human blood. In conclusion, the present study showed parity difference between the two populations of *P*. *orientalis* and that both populations have strong zoophilic behavior. Based on the presented evidences, the species is strongly implicated as a vector of kala-azar in both areas. Therefore, vector control should be a component of a strategy to manage visceral leishmaniasis in both study areas.

## Introduction

Visceral leishmaniasis (VL) in the Old World is transmitted by female *Phlebotomus* species [1] which feed on blood of vertebrate hosts to obtain protein for the maturation of their eggs [2]. During blood feeding, a sand fly may ingest the etiological agent of VL along with the blood meal from an infected host, allowing biological development of the parasite in the gut and then transmitting to another host during a subsequent feeding [3].

Based on the circumstantial evidences, *Phlebotomus orientalis* has been implicated as a vector of *Leishmania donovani* in north, northwest and southwest of Ethiopia. Demonstration of natural infection in *P*. *orientalis*, which is one of the criteria of vector incrimination [4], has been difficult except one study by Hailu et al. [5] who found *L*. *donovani* promastigotes in one *P*. *orientalis* in the lower Omo plains. Gebre-Michael et al. [6] dissected 1219 females in the Awash Valley but none was infected with *Leishmania* spp. Similar unsuccessful attempts have been made with dissection of 607 and 618 *P*. *orientalis* from Libo-Kemkem and Metema-Humera lowlands [7,8].

Blood meal identification of sand flies provides information on the type of host they are fed and also to imply the potential reservoir host (s) [9]. A good reservoir host is a major blood meal source for sand flies [4]. In malaria epidemiology, it is always common to study the proportion of mosquitoes that have fed on humans (anthropophagic) and animals (zoophagic) in order to determine the human blood index (HBI) and bovine blood index (BBI) [10]; however, this has been rarely applied in sand flies.

Identification of the blood meal source of haematophagous insects has been performed using both serological and molecular techniques. The earliest and well known serological methods were precipitin test, hemagglutination inhibition assays, counter-current immune-electrophoresis (CCIE) and at the present enzyme linked immunosorbent assay (ELISA) is widely employed [11–15]. Although these methods yielded valuable information on the identity of the hosts of many blood feeding arthropods, they are not far from limitations, such as the need to produce species-specific antibodies for each potential host and the requirement for fresh blood [16]. However, such limitations are minimized after the molecular techniques [17]. A molecular method for the identification of blood meal origin involves the amplification of either mitochondrial or nuclear DNA by polymerase chain reaction (PCR) followed by species identification using restriction digestion (PCR-RFLP), terminal restriction length polymorphisms, heteroduplex mobility assays, and sequencing. All of these approaches require relatively large amounts of PCR product and do not detect multiple blood sources in a single insect. In order to overcome these setbacks Cytochrome *b* (cyt *b*) PCR- reverse line blotting (RLB) assay was developed and used for identifying blood meal sources [18].

Few studies have been undertaken on the blood meal sources of *P*. *orientalis* in Ethiopia. Mamo [19] analyzed some blood meal specimens of this species collected from the Awash Valley using CCIE and showed that domestic animals were predominately the source of blood meal. Similarly, Gebre-Michael et al. [8] from Metema-Humera lowlands and Gebresilassie et al. [20] from Tahtay Adiyabo district, northern Ethiopia reported similar results by analyzing blood meals using ELISA.

Understanding the physiological age (parity) of a sand fly population which is established as a vector is great importance in the epidemiology of leishmaniasis, as parous female could have acquired *Leishmania* infections by feeding a reservoir or an infected person and has potential to transmit parasites [21]. High parous rates in a sand fly population imply high proportion of longer lived individuals that are potentially infected and capable of transmitting disease.

In order to control sand flies and thereby to reduce leishmaniasis, the first important step is to identify the sand fly species, which has the ability to support full development of *Leishmania* parasites and transmit the parasites to human [22]. Identification or incrimination of vector species in *Leishmania* endemic focus should fulfill at least one of the five basic vector incrimination criteria set by WHO [4]. The aim of this study was to determine the physiological age, blood meal origins and detect natural infection of *P*. *orientalis* in two ecologically distinct foci of VL in northwestern Ethiopia (Libo-Kemkem and Metema districts) where the disease is endemic.

## Materials and methods

### Study areas

The study was conducted in two ecologically distinct areas of Amhara Regional State in northwestern Ethiopia, namely Libo-Kemkem and Metema districts. The former is situated in a highland with altitude of 2000 meter above sea level (masl) and the latter in a lowland area with altitude ranges 700-750masl. The Metema—Humera plains in the north-west, bordering east Sudan, are the long-recognized VL endemic areas in the country [23]. The two regions contribute more than 60% of the disease burden in the country [24]. In addition to the lowlands, kala-azar has disseminated to the highlands of Libo-Kemkem district and neighboring areas, wherein it claimed more than 200 people [25]. Detailed description of the study areas were presented elsewhere [26].

### Sand fly collection and processing

Sand flies were collected using CDC light traps between May 2011 and April 2012 in Libo-Kemkem and October 2012 and September 2013 in Metema. For collection of flies, three villages from the highland district (namely Angot, Bura and Yifag) and three villages from the lowland district (Aftit, Bura and Mender-6) were selected. These villages were selected based on previous kala- azar cases report [25] and accessibility of the villages. In each village in both districts, three permanent sampling habitats were selected: household compound, farm field and mixed forest. The distance between the habitats ranged between 150 to 200 m. In each habitat, two light traps were deployed. During the sampling night, traps were hanged 30–50 cm above the ground from dusk to dawn. In the mornings, trapped sand flies were transported to a field laboratory where *Phlebotomus* females were categorized into different abdominal status (unfed, half-gravid, fully gravid, and freshly fed) under a dissecting microscope.

All freshly engorged *Phlebotomus* females were preserved for blood meal analysis. For this purpose, the head and tip of each fed female were severed from the rest of the body and slide-mounted separately for species identification. The rest of the body corresponding to the parts that were slide mounted was individually placed either in silica gel or absolute alcohol. The specimens preserved in silica gel stored at room temperature whereas those in absolute alcohol were stored at -20 °C until blood meal analysis carried on by either cyt *b* PCR-RLB or ELISA.

### Blood meal analysis

#### Molecular method (cyt *b* PCR-RLB)

Blood meal analysis using cyt *b* PCR-RLB was conducted following the procedure of Abbasi et al. [18] in August 2012 in the Department of Microbiology and Molecular Genetics, Hadassah Medical School, Hebrew University, Jerusalem, Israel. Analysis of blood meal by this technique was carried out for some of the specimens collected from Libo-Kemkem (May 2011-April 2012). The rest blood meal samples from both Libo-Kemkem and Metema were analyzed by ELISA at Aklilu Lemma Institute of Pathobiology, Addis Ababa University in Ethiopia.

#### DNA extraction

For DNA extraction, each blood fed specimen (thorax and abdomen of the flies) was individually placed in eppendorf tube which contained a mixture of 200 μl lysis buffer (50mM NaCl, 10mM ethylene diamine teracacetic [EDTA], 50mM Tris-HCL 1% trition X-100) and 10 μl proteniase K and homogenized with pestle. This was followed by extraction of DNA by adding 180 μl phenol and 8 μl NaCl solutions and centrifuging at 1400 rpm for about two minutes. The DNA extract was transferred into another eppendorf tube, mixed with 400 μl of ethanol, preserved at -20 °C overnight and the DNA was precipitated, after which cold centrifugation at 1400 rpm for 10 min. The DNA was dried in oven for about 10 min and the DNA pellet was re-suspended in 50 μl double distilled water (ddH_2_O).

#### DNA amplification by PCR

A 25 μl solution of PCR was prepared by mixing 5 μl DNA with 20 μl master mix (containing forward and backward primers, *Taq* DNA polymerase, dNTPs, MgCl_2_, reaction buffers and ddH_2_O). The target DNA for amplification was 344 base pairs of the conserved region of cyt *b* gene. Amplification of this region was made using the following primer pairs: forward Cyto1: 5’-CCA TCA AAC ATA TCA GCA TGA TGA AA-3’ and reverse, Cyto 2: 5’-CCC CTC AGA ATG ATA TTT GTC CTC -3’. The thermo-cycling conditions consisted of 35 cycles at 94 °C for 30 sec, 55 °C for 30 sec, and an elongation step at 72 °C for 1 min.

#### Electrophoresis

The PCR products were loaded on agarose gel (1.5%) and electrophoresed at 120 V in 1x Tris-Acetate (TAE) buffer containing 10 μl ethidium. The gels were visualized under UV light for determination of the sizes of the amplicon. The PCR amplified products were used as probes in RLB hybridization reaction.

#### RLB hybridization reaction

This procedure was done in two steps (Immobilization of oligonucleotide to the membranes and hybridization and detection) following the method described in Abbasi et al. [18].

#### Immobilization of oligonucleotide to membranes

Biodyne C nylon membrane (5.5 × 15 cm, Gelman USA) was activated by washing three times using 0.1 M HCL for 10 min. Afterward, the membrane was rinsed in ddH_2_O three times for 6 min and soaked in 10% solution of 1-ethyl-3-[3’-dimethyl amino propyl] carbodiimide (EDC) for half an hour. Then, the membrane was rinsed in ddH_2_O and left to dry. Species specific 5’-end amino linked oligonucleotide probes for human, cow, sheep, goat, camel, donkey, dog, mouse, rat, chicken, and bird (avian) developed by Abbasi et al. [18] were diluted to 5 pmol/μL and added to the membrane. The probes were linked to nylon membrane through the formation of amide bonds between the carboxyl groups on the nylon membrane and the amino groups to the oligonucleotides using a manifold blotter apparatus (Immunetics, Cambridge, MA).

#### Hybridization and detection

The nylon membrane sheet with the oligonucleotide probes was cut into strips at 90 ° to the direction of the blot; hence each strip contained all the eleven probes. Strips were placed in an incubation tray, which had eight lanes and incubated in pre-hybridization solution (2 x sodium chloride and sodium citrate [SSC] with 0.1 Sodium dodecyl sulfate [SDS]) for 30 min at 46 °C with gentle shaking. Biotinylated PCR products were denatured in water bath at 95 °C for 10 min. Hybridization of the denatured PCR products took place at the same temperature of incubation of strips for an hour. Hybridized biotinylated DNA was detected by incubating the strips in streptavidin horseradish peroxidase (HRP) for 30 min at room temperature. After washing the strips three times using 2 xSSC, 0.1%SDS, freshly prepared TMB solution (0.1mg/ml of 3, 3’, 5, 5’ tetramethylbezidine, 0.003% H_2_O_2_ in 0.1M sodium citrate [pH 5.0]), was added for chromogenic detection. After a few minutes bands were observed.

#### Serological method (ELISA)

Blood meal analysis using ELISA was performed on the remaining specimens from Libo-Kemkem and all of the specimens from Metema following the procedure of Beier et al. [13] with some modifications. Briefly, each fed fly was triturated in 1.5 ml eppendrof tube with micro tissue grinder to which 50 μl of 0.01 M phosphate buffered saline (PBS), pH 7.4, was added. The triturated sample was kept at -20 °C until analysis. Then, 50 μl of the triturate sample was diluted in carbonate/bicarbonate coating buffer (CBB) (1:50) and 50 μl of the mixture was added to wells of polyvinyl chloride, U-shaped, 96-well microtiter plates (Dynatech Laboratories, Inc., Alexandria, Va), which were covered and incubated at 4 °C overnight. Each well was washed three times with washing solution (200 μl PBS containing Tween-20). Plates were subsequently blocked by adding bovine serum albumin (BSA) and incubated for one hour at 37 °C. Each well was washed three times with washing solution (PBS-Tw-20). This was followed by the addition of 50 μl host specific conjugate (antihost IgG, Human, bovine, donkey, goat, sheep and dog) diluted 1:2000 for human, 1:250 for bovine, 1:5000 for the rest of animals) in 0.5% boiled casein containing 0.025% Tween-20. The boiled casein was prepared by boiling 5 g casein in 100 ml 0.5 N NaOH and adding 900 ml PBS (pH 7.4), 0.1 g Thimerosal (sodium ethyl mercuri thio salicylate) and 0.02 gm phenol red. After 1 h, wells were washed three times with PBS-Tween-20, and 100 μl of ABTS peroxidase substrate were added to each well. Absorbance at 405 nm was determined with an ELISA reader 30 min after the addition of substrate. Each blood meal sample was considered positive if the absorbance value exceeded the mean plus three standard deviations of the mean of three negative controls and also by observing color change (green color). Negative controls were prepared using unfed insectary female *P*. *orientalis* from ALIPB. Positive controls were blood specimens of the six hosts.

The anti-immunoglobulin antisera were pre-screened to verify their antigentic specificity by reactions with blood meal samples of the six hosts. Cross- reaction was noted only between anti-goat and anti-sheep antisera and the result of these (goat and sheep) reported as one, i.e. goat/sheep.

### Dissection of sand flies for determination of physiological age and detection of natural infection

Unfed females of *Phlebotomus* spp. were dissected for determination of parous state. Flies were first washed twice in 2% savlon in saline and once in sterile physiological saline. Then, each female fly was dissected in a drop of physiological saline on a glass slide under a dissecting microscope. The ovaries were pulled out along with the gut of the fly, and then covered with a small cover slip for examination under the microscope. Parous females were distinguished from nulliparous by the presence of granules in the accessory glands [21] as well as ovarian features described by Gebre-Michael et al. [27]. The gut of parous female was instantaneously examined with 10 x and 40 x objectives of a compound microscope for the presence of flagellated parasites. The guts of half and fully gravid females *Phlebotomus* were also examined. After microscopic examination, the guts of parous, half-gravid and gravid females in saline were transferred to 70% alcohol for further detection of parasites using molecular method if in case infection might have been overlooked.

#### Detection of *Leishmania* parasites using molecular method

Four procedures were followed to detect *Leishmania* parasites in the preserved guts of dissected and undissected sand flies. These were DNA extraction, DNA amplification, gel electrophoresis and sequencing of DNA samples to identify the species of *Leishmania* [28]. DNA extraction and gel electrophoresis steps were similar to that of the steps used in blood meal analysis using molecular method. However, DNA amplification step was quite different as it targeted different DNA base pairs and used different primers to that of the blood meal analysis. The target DNA for amplification was 346 bp of the whole internal transcribed spacer (ITS) in the ribosomal operon. The DNA was amplified using the primers LITSV (5’-ACACTCAGGTCTGTAAC-3’) and LITSR (5’-CTGGATCATTT-TCCGATG-3’). The positive controls were *L*. *donovani*, *L*. *major* and *L*. *aethiopica* whereas the negative control was distilled water.

### Data analysis

The data were entered in SPSS sheet and statistical analysis was made with SPSS IBM version 20.0 (Armonk, NY, IBM Corpn.). Monthly variation in parity rate of *P*. *orientalis* was determined by Chi-square test. The human blood index (HBI) and bovine blood index (BBI) were calculated as the proportion of the sand flies fed on either human or bovine blood meals out of the total blood meals determined [10].

## Results

### Abdominal status of *Phlebotomus* spp. in both areas

A total of 1314 female *P*. *orientalis* were collected from Libo-Kemkem, and the majority, 958 (72.9%) were unfed, followed by 205 (15.6%) freshly fed and 151 (11.5%) gravid or half-gravid. In Metema, of a total 484 *P*. *orientalis* females examined, 213 (44.0%) were unfed, 192 (39.7%) freshly fed and the rest 79 (16.3%) gravid or half-gravid (Table 1).

**Table 1:** Abdominal status of female *Phlebotomus* species sampled from Libo-Kemkem and Metema.

Species	No. collected		No. unfed (%)		No. gravid (%)		No. fresh fed (%)	
	Libo-Kemkem	Metema	Libo-Kemkem	Metema	Libo-Kemkem	Metema	Libo-Kemkem	Metema
*P*. *orientalis*	1314	484	958(72.9)	213(44)	151(11.5)	79(16.3)	205(15.6)	192(39.7)
*P*. *rodhaini*	1	46	1 (100)	39(84.8)	0	7(15.2)	0	0
*P*. *papatasi*	0	3	0	1(33.3)	0	2(66.7)	0	0
*P*. *duboscqi*	0	2	0	1(50)	0	1(50)	0	0
*P*. *bergeroti*	0	1	0	0	0	1(100)	0	0
Total	1315	536	959 (72.9)	254 (47.4)	151 (11.5)	90 (16.8)	205 (15.6)	192(35.8)

### Parity rates

**Fig 1:**
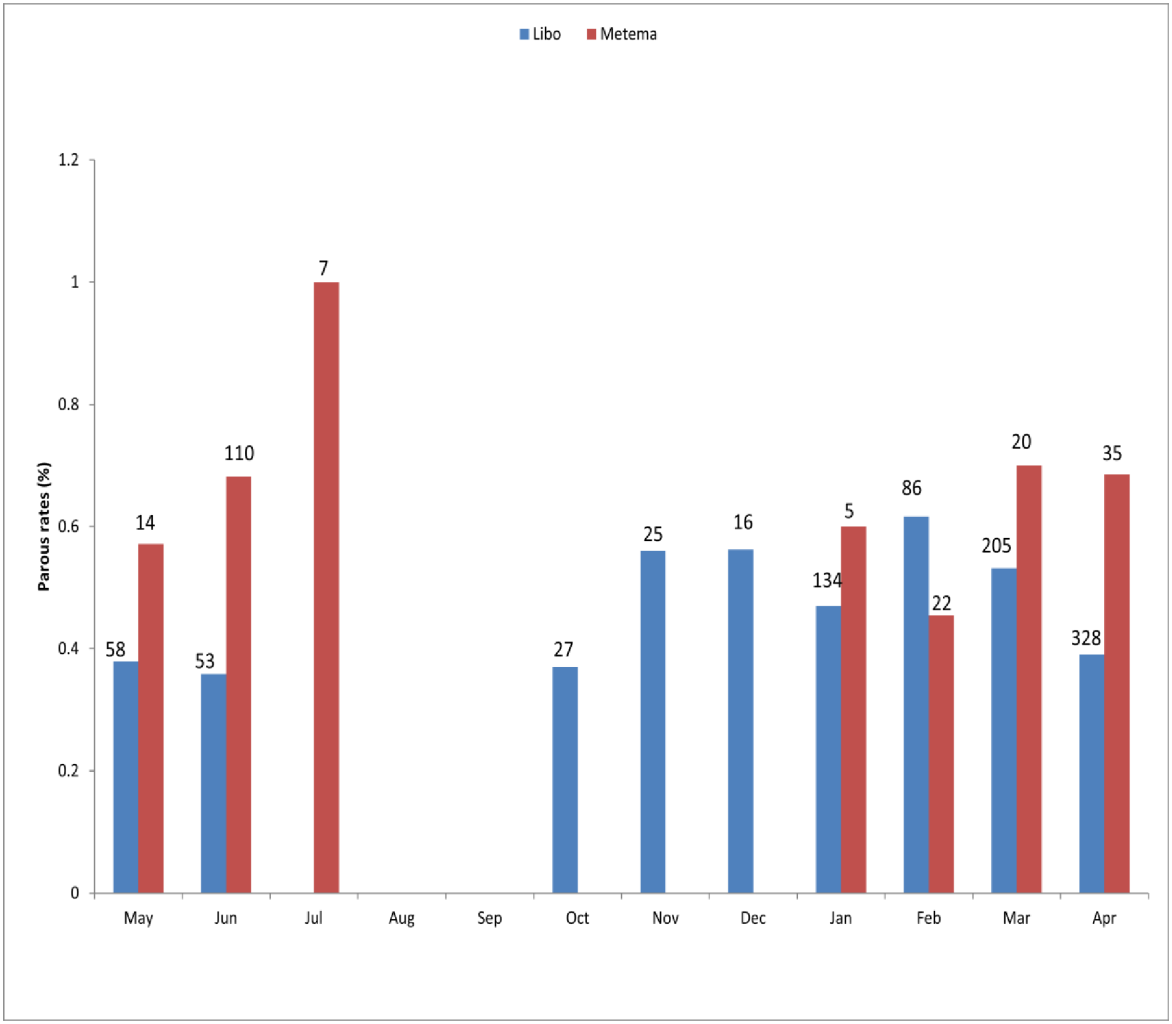
Monthly parous rate of *P*. *orientalis* from Libo-Kemkem (May 2011-April 2012) and Metema (October 2012- September 2013) districts. N. B. number above the bar indicates the total number of *P*. *orientalis* dissected per month.

In Libo-Kemkem, a total of 936 unfed *P*. *orientalis* were dissected and the parity rate was 45.6% (n = 427), ranging from 37.0 in October to 61.6% in the middle of the dry season (February) with obvious seasonal trend with statistical significance was observed among the months (χ2 = 23.8, df = 9, P<0.05). In Metema, out of a total of 213 unfed *P*. *orientalis* dissected the mean parous rate was 66.2% (n = 141), ranging from 0 during the early dry season (October-December) to 100% at the beginning of the rainy season (July), thus showing clear seasonal pattern (Fig 1), however no statistical significance was observed among the months (P>0.05). From the small number of *P*. *rodhaini* dissected (n = 39) in Metema, 46.1% (n = 18) were parous.

### Blood meal identification based on Cyt *b* PCR-RLB

**Fig 2:**
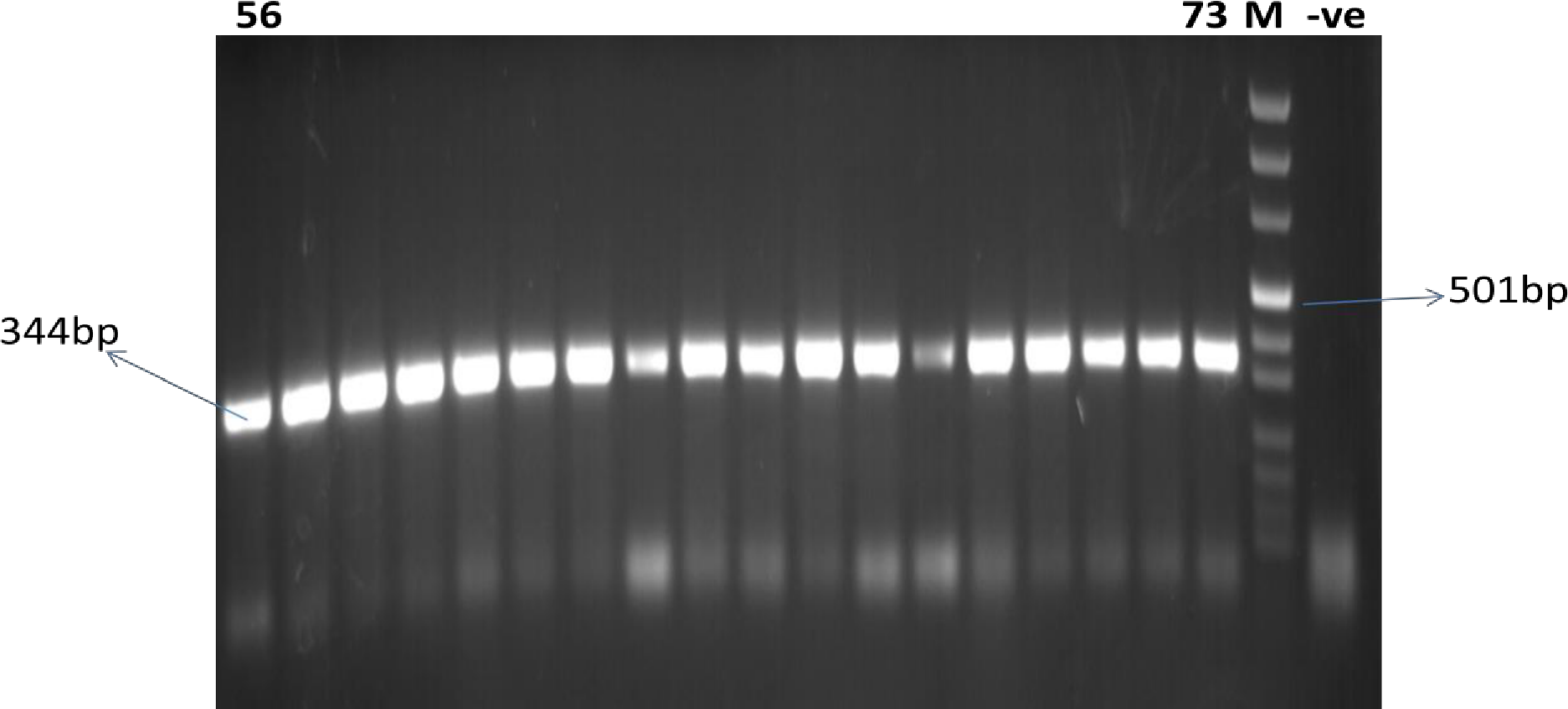
Gel image of cyt *b* PCR targeting DNA extracted from wild caught blood fed *P*. *orientalis*. Lanes 56 to 73 are PCR products of blood fed sand fly amplified at the region of cyt *b*. M is DNA marker.–ve, negative control.

**Fig 3:**
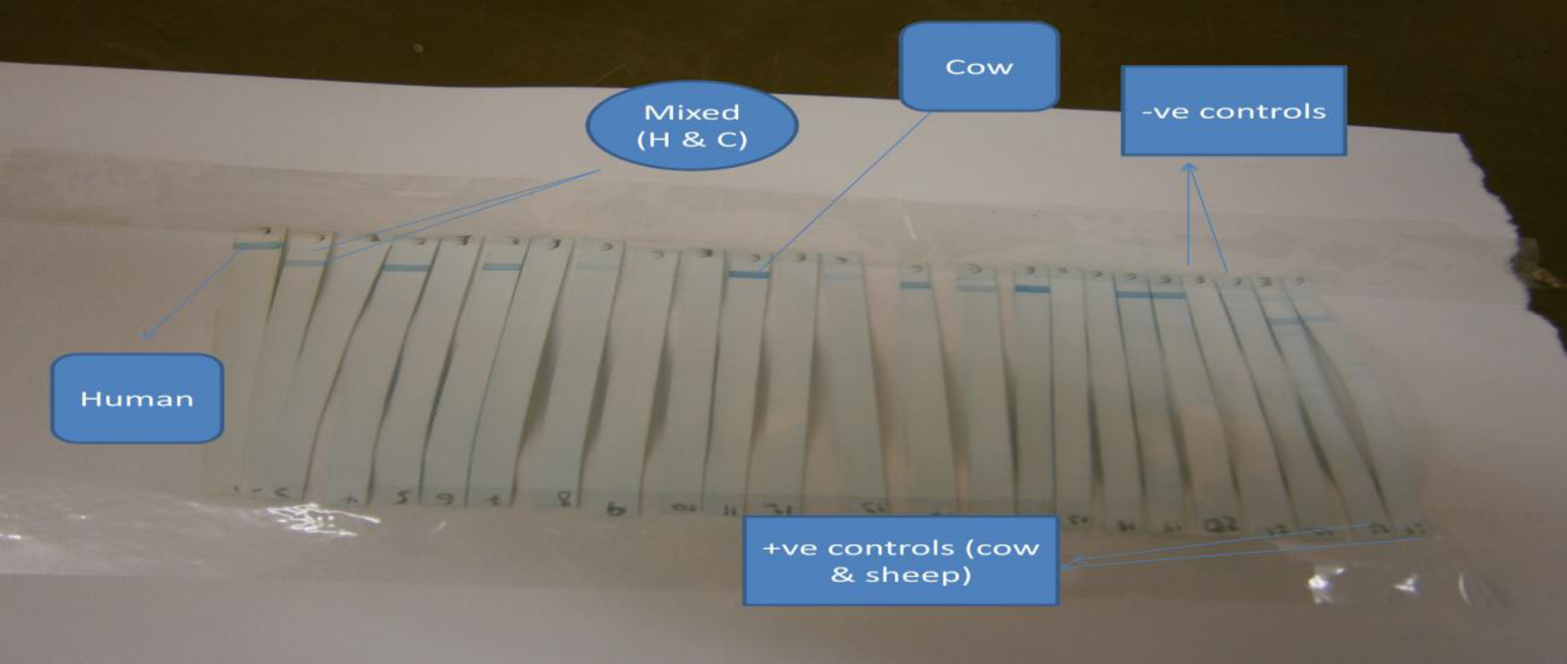
Reverse line blotting results of cyt *b* PCR products from wild caught blood fed *P*. *orientalis*. H = human, C = cow.

A total of 216 blood fed females of *P*. *orientalis* were collected from May 2011 to April 2012 in Libo-Kemkem. Out of these, 115 flies were analyzed using cyt *b* PCR-RLB to identify the blood meal sources. The remaining blood samples were analyzed by ELISA. Of the 115 *P*. *orientalis*, 113 (98.3%) were positive to cyt *b* PCR (Fig 2) and were used for blood meal identification using RLB and the results after immobilization, hybridization and chromogenic detection (RLB) revealed that 75 (66.4%) were of cow origin and 6 (5.3%) were from human origin. Mixed blood meal for human-cow was detected in 18 (15.9%) and the rest 14 (12.4%) were unidentified (Table 2 and Fig 3).

**Table 2:** Sources of blood meals of *P*. *orientalis* sampled from Libo-Kemkem and Metema districts and identified by cyt *b* PCR-RLB and ELISA.

Blood meal sources	District				Total (%) 491
	Libo-Kemkem			Metema	
	RLB = 113	ELISA = 101	Combined = 214	ELISA = 277	
Bovine	75 (66.4)	70 (69.3)	145 (67.8)	138 (49.8)	283 (57.6)
Human	6 (5.3)	2 (1.9)	8 (3.7)	16 (5.7)	24 (4.9)
Donkey	0	0	0	15 (5.4)	15 (3.1)
Dog	0	0	0	4 (1.4)	4 (0.8)
Sheep/Goat*	0	1 (0.99)	1 (0.5)	4 (1.4)	5 (1.0)
Human-Bovine	18 (15.9)	11 (10.9)	29 (13.6)	5 (1.8)	34 (6.9)
Human-Donkey	0	1 (0.99)	1 (0.5)	9 (3.2)	10 (2.0)
Human-Dog	0	0	0	3 (1.1)	3 (0.6)
Bovine-Donkey	0	1 (0.99)	1 (0.5)	6 (2.2)	7 (1.4)
Bovine-Dog	0	1 (0.99)	1 (0.5)	9 (3.2)	10 (2.0)
Bovine-Sheep/Goat	0	0	0	2 (0.7)	2 (0.4)
Bovine-Donkey-Dog	0	0	0	1 (0.4)	1 (0.2)
Unidentified	14(12.4)	14 (13.9)	28 (14.0)	65 (23.5)	93 (18.9)

* Since there was cross-reaction between the two antisera the results are presented as goat/sheep

### Blood meal identification based on ELISA

In Libo-Kemkem, of the remaining 101 *P*. *orientalis* tested by ELISA, blood of five animals was identified. These were bovine blood alone 69.3% (n = 70), human alone 1.9% (n = 2) and goat/sheep alone 0.99% (n = 1). Mixed blood meals were also identified that included; 10.9% (n = 11) for human-bovine blood, 0.99% (n = 1) for human-donkey blood, 0.99% (n = 1) for bovine-donkey blood and 0.99% (n = 1) for bovine-dog blood. The remaining 13.9% (n = 14) blood meal samples were unidentified (Table 2).

The HBI (including mixed feeding) of *P*. *orientalis* in Libo-Kemkem based on both methods of analysis (cyt *b* PCR-RLB and ELISA) was a little over 0.17 whereas the BBI (including mixed feedings) was 0.82, showing its higher predilection for cattle.

In Metema, a total of 277 blood fed females *P*. *orientalis* were collected during the course of the study period and were all analyzed by ELISA. The pattern for single hosts was: 138 (49.8%) for bovine blood, 16 (5.7%) for human blood, 15 (5.4%) for donkey blood, 4 (1.4) for dog blood, 4 (1.4%) for sheep/goat blood. Mixed blood meals from either two hosts (six) or three hosts (one) were also identified that included: 9 (3.24%) of bovine-dog, 9 (3.24%) of human-donkey, 6 (2.2%) of bovine-donkey, 5 (1.8%) of bovine-human, 3 (1.1%) of human-dog, 2 (0.7%) of bovine-sheep/goat and including one tri-host (0.36%) from bovine-donkey-dog. Specimens for 65 (23.5%) blood meals remained unidentified (Table 2).

The HBI for *P*. *orientalis* (including the mixed blood feedings) in Metema was 0.12 while the bovine blood index (including the mixed feedings) was 0.58 showing a predilection for cattle, though much lower than in Libo-Kemkem.

### *Leishmania* detection based on dissection and molecular method

In Libo-Kemkem, the guts of 578 females *P*. *orientalis* (427 parous and 151 gravid) were observed for detection of natural infection (Table 3) and none was positive. Similar results were also obtained from 251 *Phlebotomus* spp. (141 parous and 79 gravid *P*. *orientalis*, 18 parous and 7 gravid *P*. *rodhaini*, 1 parous and 2 gravid *P*. *papatasi* and 1 parous and 1 gravid *P*. *duboscqi*) in Metema.

**Table 3:** Guts dissection results in Libo-Kemkem and Metema districts.

Species	Libo-Kemkem			Metema			Total dissected (% infection)
	No. parous Dissected	No. gravid dissected.	No. infection (%)	No. parous dissected	No. gravid dissected	No. infection (%)	
*P*. *orientalis*	427	151	0	141	79	0	798 (0)
*P*. *rodhaini*	1	0	0	18	7	0	26 (0)
*P*. *papatasi*	0	0	0	1	2	0	3 (0)
*P*. *duboscqi*	0	0	0	1	1	0	2 (0)
*P*. *bergeroti*	0	0	0	0	1	0	1 (0)
Total	428	151	0	161	90	0	830 (0)

**Fig 4:**
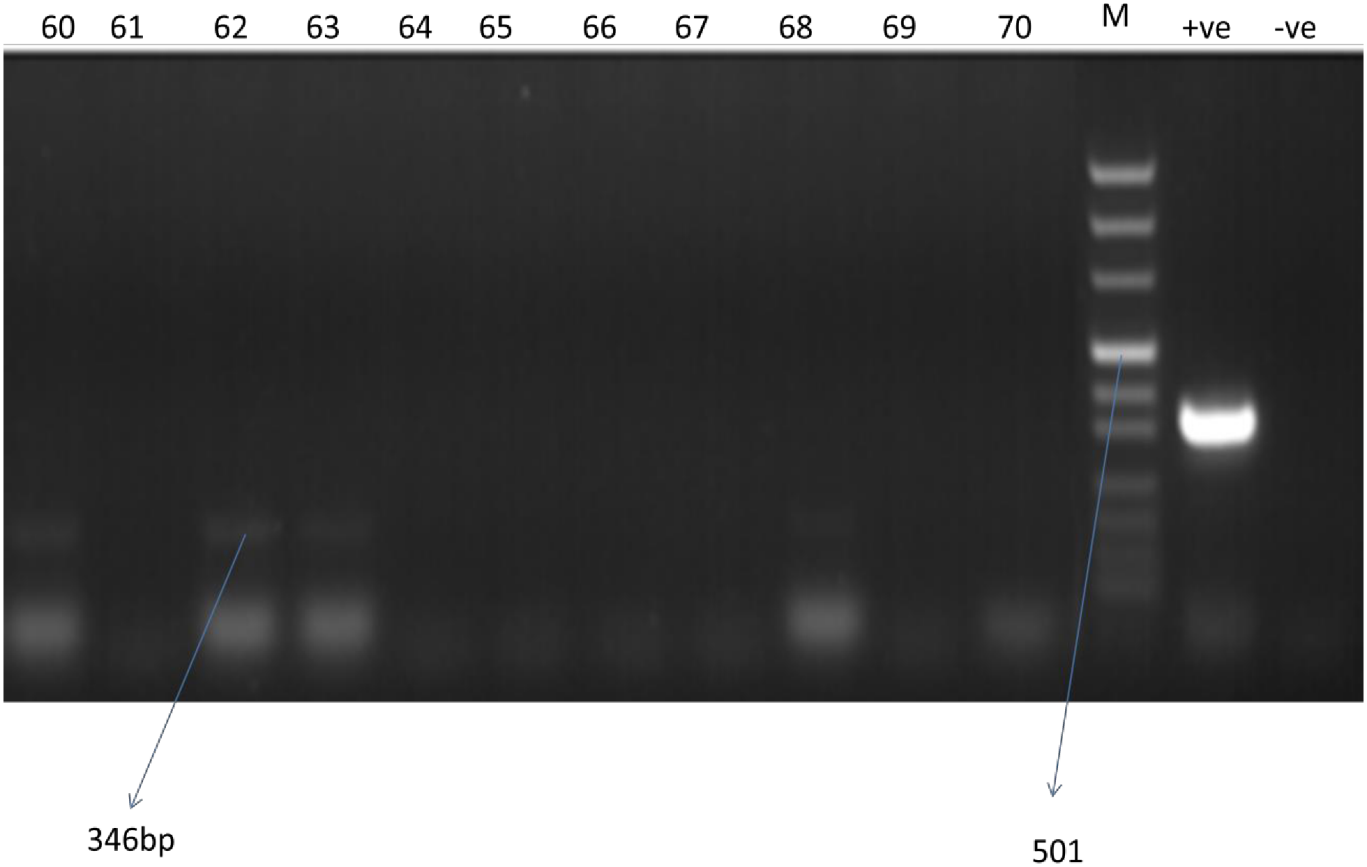
Agarose gel electrophoresis of PCR amplification of extracted from wild caught *P*. *orientalis*; Lanes 60 to 70 PCR products sand fly amplified for ITS region. M is DNA marker. +ve- positive control, -ve- negative control.

Of the PCR tested 347 *P*. *orientalis* from Libo-Kemkem, 10 (2.8%) were positive to *Leishmania* spp. (Fig 4), however, sequencing did not give positive result due to low concentration of DNA.

## Discussion

In this study the parity rate, blood meal sources and the status of *Leishmania* infection in *P*. *orientalis* and other *Phlebotomus* species in two geographically separate areas were investigated as part of an epidemiological study on the ecology of leishmaniasis in the northern and north west of Ethiopia. The findings are additional evidences for the role of *P*. *orientalis* in the transmission of leishmaniasis.

Recognition of parous females of sand flies has epidemiological significance as these flies must survive at least one oviposition cycle to be potentially infective with *Leishmania* parasites [29]. A focus that sustains a significant percentage of parous sand flies is expected to contain long lived individuals than a focus with low parity rate. In this study, the overall parous rate of *P*. *orientalis* was lower in Libo-Kemkem than Metema. This indicates that highland population of *P*. *orientalis* are short-lived, with most failing to survive long enough to take a second blood meal, presumably dying during or immediately after oviposition in comparison with the lowland population of the species. Similar observation was also noted by Anez et al. [30] in Venezuela. However, parous rates of the species in both populations were higher than that of previous observations reported from these areas [7, 8]. Such discrepancy might be due to seasonal differences as earlier investigations were done during a brief period of time in both study areas and relatively smaller numbers of females were dissected in Metema in the present study.

This is the first attempt to describe the natural blood meal sources of *P*. *orientalis* in Libo-Kemkem where five hosts (single and mixed) were identified as sources of blood meals for *P*. *orientalis* of which bovine was the most preferred host. The results indicated that humans were comparatively less attractive to this species and thus, the human blood index (0.17) was much lower than the bovine blood index (0.82), exhibiting the zoophily. A similar result was also obtained from Metema, however the BBI was lower. Such differences might be due to the lowland population of *P*. *orientalis* having more access to other hosts such as goat, sheep, and donkey as compared to the highland population, where most goats and sheep were kept inside human dwellings during the night. These data from both areas agree with a previous observation in Metema by Gebre-Michael et al. [8] who showed the zoophilic nature of *P*. *orientalis*, cattle being the major host (92%) and much higher than the present study. Study on host choice conducted recently in Sheraro, north Ethiopia, hascorroborated the present observation where cattle were found to attract the highest number of *P*. *orientalis* [20]. In contrast, a study on host attractiveness in a limited number of animal hosts (dog, mongoose, Nile rat and genet) to *P*. *orientalis* in eastern Sudan, showed that dog baited trap significantly attracted the highest number of *P*. *orientalis*, though no other domestic animals were used in the experiment [31]; there is yet no results available on blood meal analysis of *P*. *orientalis* from Sudan.

The driving force behind the tendency of *P*. *orientalis* for cattle to blood meals other hosts in the present study is not known; however from previous knowledge on host preferences of other haematophagous insects, different explanations can be given. One of these reasons could be the relative abundance of the hosts which might determine the host preference of vector species [32]. In both study areas large numbers of cattle with small number of goats, sheep, donkeys and dogs were present outside of human dwellings and these animals were guarded by two to three adult men during the night. The other reason could be difference in body size of the animals. This difference in size in turn results in variation in the amount of CO_2_ released by the animals [33]. Thus, larger animals like cattle could be more attractive than the rest, although some authors suggested that animal size had no any effect on host selection by sand flies in the cases of *Lu*. *olmeca*, *Lu*. *panamensis* and *Lu*. *vespertilionis* [34].

The role of cattle in the epidemiology of VL is controversial. In India, Barnett et al. [35] concluded that owning a large number of cattle is a risk factor for VL transmission. A similar result was also reported by Bucheton et al. [36]. These studies hypothesize the presence of plethora cattle in the areas might serve as blood meal source for the vector species and also the byproduct of these animals could be an important source of food for sand fly larvae and as a result of which the abundance of the vector species increases thereby increasing transmission of the disease. In contrast, Bern et al. [37] reported that increasing cattle density around house decreased kala-azar transmission by sand flies in Bangladesh. It has been suggested that the proximity of cattle to human dwellings diminish kala-azar transmission by enabling vector species to feed preferentially on animals, not susceptible to leishmaniasis (dead end host), thereby decreasing vector-human contact (zooprophylaxis effect) [38]. However, the role of cattle in both our study areas other than serving as main blood meal sources is not clear.

The detection of other blood meal hosts, though at lower proportion, might indicate the opportunistic behavior of *P*. *orientalis*, enabling it to feed on those readily available hosts. Such behavior is common in a number of species observed elsewhere [19, 39–42].

In both areas, particularly in the lowland region, a considerable proportion of *P*. *orientalis* analyzed had contact with more than one host (mixed feeding) during a single gonotrophic cycle. Of these, 13.7% had double meals and 0.4% contained triple meals, presumably on the same night. Such mixed blood meals are commonly observed in other sand flies [40, 41]. The exact cause of mixed feeding for this and other species of sand flies in nature is not apparent, however, it is believed to be a consequence of interrupted feedings due to difficulties faced while they engorge on a single host-due to host defensive behavior [42, 43], or when the fly encounters an unnatural host forcing it to divert to another nearby host before repletion from the first host, or the fly might be infected by *Leishmania* parasites promotes multiple feeding [44].

The numbers of unidentified blood meals in both areas were considerable: 28 (14.0%) in Libo-Kemkem and 65 (23.5%) in Metema. The failure to determine the blood meal sources of *P*. *orientalis* may have occurred as a result of either not using probes/antisera of both large (e.g. warthog) and small (e.g. murines and canines) wild mammals which were seen frequently in the study areas especially in Metema. It could also be due to the poor quality of the blood meal resulting from enzymatic degradation of the blood.

Owing to the populations of *P*. *orientalis* in both localities having a strong predilection for cattle, treating of these animals by topical application of insecticides [45] or systematic insecticides such as ivermectin [46] may help in alleviating the burden of the disease in the study areas. However, proper evaluations on these potentialities are required.

Identification of vectors and determination of natural infection rates with *Leishmania* spp. in wild flies are important for definition of risk factors and control of leishmaniasis [47]. In the current study, no natural infection was detected in dissected *P*. *orientalis* and *other Phlebotomus*. In Libo-Kemkem a few specimens were found with *Leishmania*; DNA sequencing, however, did not produce any results. Absence of promastigotes in the gut of dissected *P*. *orientalis* is not uncommon in Ethiopia. Ashford et al. [48] dissected a total of 1006 females of this species in Belessa and found no infection. Previous studies in Libo-Kemkem [7], Humera and Metema districts reported [8]. Natural infection rates in phlebotomine sand flies are usually low, and it is sometimes necessary to dissect thousands of sand flies to find an infected specimen [49]. This might be due to deficiencies in sampling, intensity of the disease or short life span of the vector. In previous studies by Gebre-Michael et al. [7, 8], the majority of dissected flies were nulliparous, while the parous rates were only 30–34% in both the highland (Libo-Kemkem) and lowlands (Humera and Metema). On the other hand, Hailu et al. [50] found natural *L*. *donovani* infection in one of 70 (1.4%) dissected female *P*. *orientalis* in southwest Ethiopia, while Ashford et al. [51] detected four infected among 48 *P*. *orientalis* (8.3%) during an epidemic of the disease in south Sudan.

Although natural infection was not detected in either methods in the present observations, *P*. *orientalis* appears to be the principal vector of *L*. *donovani* in northwestern Ethiopia mainly by the following reasons; firstly, *P*. *orientalis* is the only species in the genus *Phlebotomus* which is found in large numbers in both areas [23]; second, in a recent study on susceptibility of *P*. *orientalis* (colony of Libo-Kemkem population) to *L*. *donovani* showed that this species supports full development of *L*. *donovani* promastigotes in the gut and the parasite colonized in the anterior parts of the midgut and the stomodeal value of the flies [52] and lastly, *P*. *orientalis* is a proven vector in neighboring Sudan [51, 53].

In conclusion, the present study shows that the difference in the parity rates between the highland and lowland populations of *P*. *orientalis* being higher in the lowland population. It also indicates that both populations of *P*. *orientalis* have strong zoophilic behavior. Furthermore, in both areas the species is strongly implicated as a vector of kala-azar. Therefore, in the future vector control should be one of the approaches for the management of leishmaniasis in the two foci.
